# Effect of chemicals on the phase and viscosity behavior of water in oil emulsions

**DOI:** 10.1038/s41598-023-31379-0

**Published:** 2023-03-12

**Authors:** Masoud Shafiei, Yousef Kazemzadeh, Dmitriy A. Martyushev, Zhenxue Dai, Masoud Riazi

**Affiliations:** 1 Enhanced Oil Recovery Research Center, School of Chemical and Petroleum Engineering, Shiraz University, Shiraz, Iran; 2 Department of Petroleum Engineering, Faculty of Petroleum, Gas, and Petrochemical Engineering, Persian Gulf University, Bushehr, Iran; 3 Department of Oil and Gas Technologies, Perm National Research Polytechnic University, Perm, 614990 Russia; 4 College of Construction Engineering, Jilin University, Changchun, China

**Keywords:** Engineering, Chemical engineering

## Abstract

Due to population growth, the need for energy, especially fossil fuels, is increased every year. Since the costs of exploring new reservoirs and drilling new wells are very high, most reservoirs have passed their first and second periods of life, and it is necessary to use EOR methods. Water-based enhanced oil recovery (EOR) methods are one of the popular methods in this field. In this method, due to the possibility of emulsion formation is high, and by creating a stable emulsion, viscosity and mobility improved. In this study, the parameters affecting the stability and viscosity of the emulsion have been investigated step by step. In the first step, 50% (v/v) of water has been selected as the best water cut. The type of salt and its best concentration was evaluated in the second step by measuring the average droplets size. The third step investigated the effect of SiO_2_ nanoparticles and surfactant (span80) on emulsion stability and viscosity. According to the results, the best amount of water cut was 50% due to the maximum viscosity. In salts the yield was as follows: MgCl_2_ > CaCl_2_ > MgSO_4_ > Na_2_SO_4_ > NaCl. The best yield was related to MgCl_2_ at a concentration of 10,000 ppm. Finally, it was shown that the synergy of nanoparticles and surfactants resulted in higher stability and viscosity than in the case where each was used alone. It should be noted that the optimal concentration of nanoparticles is equal to 0.1% (w/w), and the optimal concentration of surfactant is equal to 200 ppm. In general, a stable state was obtained in 50% water-cut with MgCl_2_ salt at a concentration of 10,000 ppm and in the presence of SiO_2_ nanoparticles at a concentration of 0.1% and span 80 surfactants at a concentration of 200 ppm. The results obtained from this study provide important insights for optimal selection of the water-based EOR operation parameters. Viscosity showed a similar trend with stability and droplet size. As the average particle size decreased (or stability increased), the emulsion viscosity increased.

## Introduction

The need for energy, especially fossil fuels, is increasing every year due to population growth. Exxon Mobil forecasts a 25% increase in energy demand by 2040 compared to 2018. Moreover, because most of the world's reservoirs are in the second and third periods of their life, the need to increase the efficiency of reservoirs and the use of enhanced oil recovery methods are strongly felt^1,2^. One of the common EOR methods in oil reservoirs is water-based methods such as injection of nanoparticles, surfactants, injection of water with different salinities, injection of polymers, or a combination of these^3^. Injection of these chemicals into the reservoir can create the conditions for emulsion formation and prevent the viscous fingering phenomenon. So the fluid front will be almost straight. Stable emulsions can significantly increase oil production from reservoirs^4–7^. Pei et al. compared flooding with or without a stabilized emulsion with nanofluids in their study. They found that injection of stable emulsion by nanofluid with two mechanisms increases the displacement efficiency: (1) blocking high permeability pathways in which water flows. (2) Mobilized trapped oil. They also stated that stability increases with increasing surfactant concentration when the silica nanoparticle concentration is 0.4%^8^. Emulsion formation is possible in all production stages (from inside the reservoir to pipelines and even drilling fluid processing)^9^.

Emulsions used in EOR are often provided by an emulsifier that reduces the interfacial tension by being present at the interface of the two phases of oil and water, thus increasing the stability and ease of emulsification^10^. Surfactants can be used to facilitate the formation and increase the stability of emulsions. These materials can change the stability of the emulsion by reducing the interfacial tension in systems where only surfactants were used for emulsion stability. Due to the sensitivity of surfactants to temperature and their high adsorption on the reservoir rock, their use as an effective fluid for the EOR process has many limitations^11,12^. Nanoparticles have been proposed to overcome these problems at the reservoir scale. The synergy of the surfactants with the nanoparticles creates a mechanical barrier that prevents the emulsion droplets from coalescence^13,14^. In addition, it can improve the EOR process by increasing the thermal stability and viscosity of the emulsion^15–17^. When nanoparticles and surfactants are used for the emulsion formation, the stability is moreover than in the case where only one of these two materials is used, and the consumption of surfactant is significantly reduced^18–20^.

On the other hand, the presence of nanoparticles due to their environment-friendly properties and safety for human health has attracted the desire to use them more. In conventional emulsions, due to their amphipathic properties, stability and emulsification depend on the arrangement of surfactants at the oil–water interface. While in Pickering emulsions, one or more layers are formed and stabilized as a film by adsorbing nanoparticles on the interface^21–24^. So far, many nanoparticles have been investigated for the stability of emulsions alone or in combination with surfactants and polymers, such as calcium oxide, titanium oxide, and iron oxide, which has increased the stability of various emulsions^25,26^. Among nanoparticles, silicon oxide nanoparticles have received more attention than other materials used in EOR, such as surfactants and polymers in the oil industry, due to their safety, availability, and environmentally friendly properties^27^. The efficiency of improving the stability of emulsions by nanoparticles depends on the nanoparticle size, surface properties, and nanoparticle concentration. The nanoparticles can mechanically provide the repulsive force necessary for the coalescence of droplets by adsorbing on the interface, which increases the stability of emulsions and reduces the number of droplets^28,29^. The presence of nanoparticles increases the mobility ratio of the injected fluid due to an increase in viscosity, making it a more suitable fluid for the EOR process. In addition, nanoparticles can reduce interfacial tension, change wettability, as well as increase emulsification and emulsion stability, and for these reasons, they can increase production capacity and reduce residual oil saturation^30^. In 2017, Kumar et al. investigated the effect of nanoparticles, surfactants, and polymers on the emulsification process and emulsion stability. They stated that the synergistic effect of sodium dodecylbenzene sulfonate and SiO2 nanoparticles with polymer reduces droplet aggregation and interfacial tension, which improves the rheological behavior of the emulsion^14^. Laboratory studies conducted by Chen et al. In 2018 have shown that nanoparticles and surfactants can reduce droplet size by reducing interfacial tension to a specific concentration and preventing creaming and coalescence processes, which leads to greater emulsion stability^31^. Ansari et al. In 2020, investigated the stability of emulsions with surfactants and nanoparticles. They stated that the presence of nanoparticles and surfactants increases the stability of emulsions and reduces the aggregation of droplets in emulsions^32^. The use of nanoparticle-stabilized emulsions was also investigated in Saskatchewan's pilot experiments to enhance oil recovery due to the higher viscosity of emulsions. The results showed that the recovery factor increased compared to conventional water injection^33–35^. In 2018, Mohsin et al. examined the effect of various nanoparticles and surfactants. They found that cationic and non-ionic surfactants can produce stable emulsion at temperatures of 60 and 40 °C, respectively, but anionic surfactants do not have this ability. SiO_2_ and Al_2_O_3_ nanoparticles can increase this stability^34^.

As well as these two parameters, the concentration and type of salts in the aqueous phase will affect the stability of the emulsion as well. Several studies have demonstrated that the three ions, Mg^2+^, Na^+^, and Ca^2+^, which are called "potential-determining ions", have a positive impact on oil recovery. These ions can change the water and oil system due to their ionic radius and charge and generally good charge density. In addition to the type of ion, the concentration of ions is also very important. Interfacial tension and droplet size at different concentrations show different trends. The concentration of these active ions must be at an optimal level to be able to bring the polar components of the oil to the interface between water and oil and change the arrangement of molecules in such a way as to reduce interfacial tension and reduce capillary force and increase oil recovery^36^. Emulsion formation and stability is sometimes enabled by the presence of different ions in the formation water or injected water. Emulsion formation sometimes reduces production and sometimes improves production from oil reservoirs. Therefore, it is important to study the effects of ions on emulsion formation, stability, and viscosity^37–39^. In 2020, Hunter et al. assessed the stability of water-in-oil emulsions using NaCl salt and found that by increasing the NaCl concentration to the threshold concentration, the emulsion stability would increase, but after that, no stability increase would be observed^40^.

**Figure 1:**
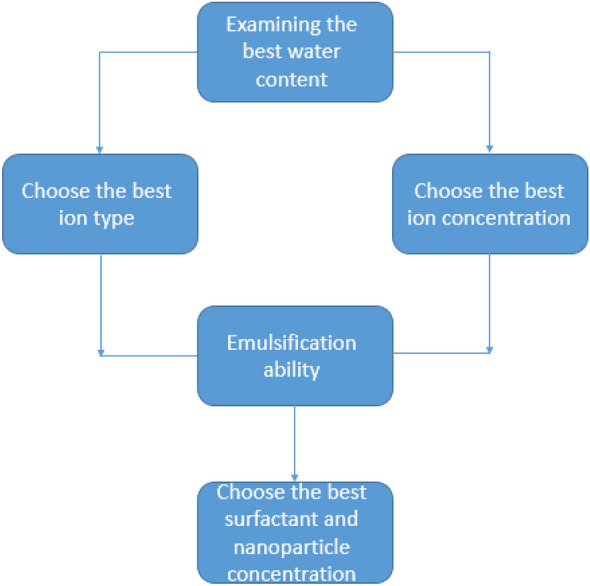
Flow work of research.

In general, the stability parameters in Pickering emulsions are more complex than in conventional emulsions. In this type of emulsion, in addition to parameters such as salt concentration and type^41–43^, water phase pH^44–49^, temperature^50–52^, nanoparticle concentration^53–58^, nanoparticle wettability^59^, and nanoparticle morphology^50,60–64^ affect the stability of the emulsion. In this paper, four general parameters were investigated: (1) the effect of water content (water cut), (2) the effect of salt type and concentration, (3) the effect of surfactant, (4) the effect of nanoparticles on the stability and rheological behavior of emulsion. In this research, four work steps were studied as below flow work: (1) obtaining the appropriate percentage, (2) obtaining the type and concentration of salt, (3) investigating emulsification ability, (4) examining the best concentration of surfactant and nanoparticles by bottle test and viscosity test (Fig. 1).

## Material and method

### Materials

**Figure 2:**
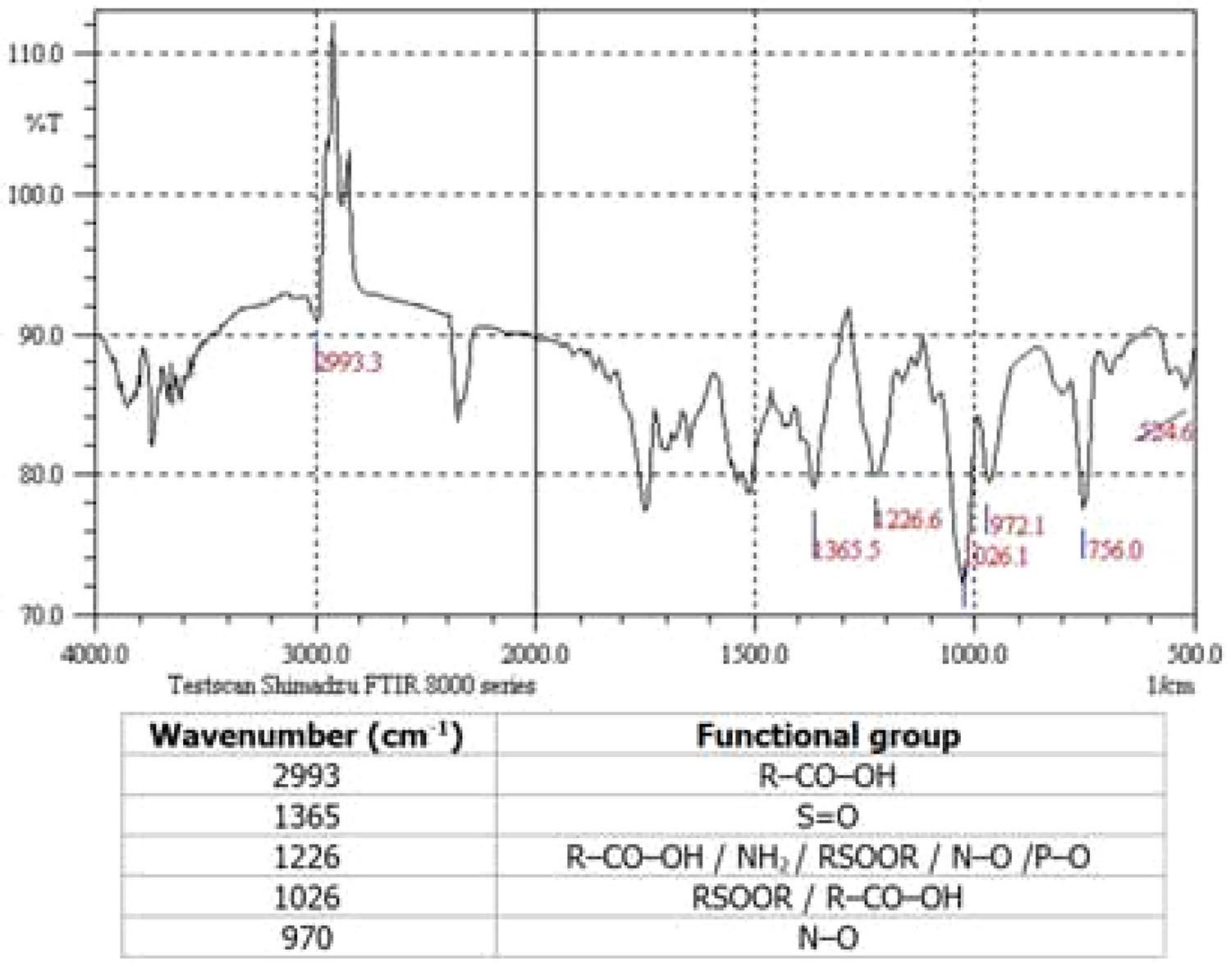
FTIR analysis of crude oil.

**Figure 3:**
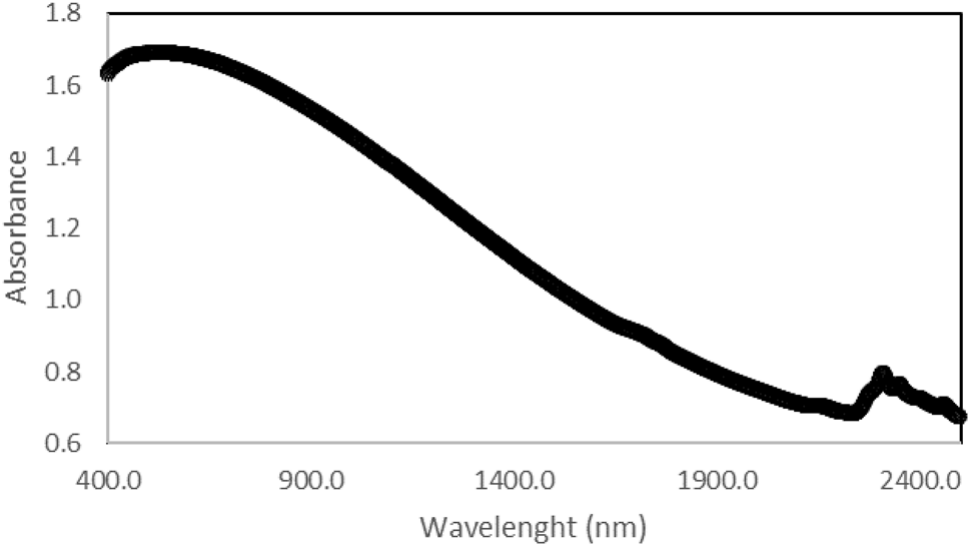
NIR analysis of asphaltene extracted from the tested oil.

The crude oil used in this study was collected from one of the oil fields in southern Iran, the characterization which is given in Tables 1, 2 and Figs. 2, 3.

**Table 1:** Characteristics of the oil.

Densit g/cm^3^	Viscosity at 25 °C cP	SARA			
		Saturates	Aromatics	Resins	Asphaltenes
0.80	180.63	40.85%	48.79%	2.61%	7.71%

**Table 2:** Constituents of the crude oil.

Components	C_2_	C_3_	iC_4_	nC_4_	iC_5_	nC_5_	C_6_	C_7_	C_8_	C_9_	C_10_	C_11_	C_12+_
Mole %	0.49	0.73	0.47	0.94	0.55	0.55	7.64	6.18	5.44	4.90	4.71	4.14	63.26

The API of this oil is 21, and the constituents of the oil are in Table 2.

The present study involved preparing a brine using various types of salts in order to form water–oil emulsions. The emulsions were prepared using high purity salts from a German company to ensure that the salts dissolved in water were pure. The salts used include monovalent salts of sodium chloride and divalent salts of magnesium chloride, sodium sulfate, magnesium sulfate, and calcium chloride. Table 3 shows the characteristics of the salts.

**Table 3:** Salts characteristics.

Salt	MW/g mol^−1^	Purity%	Chemical formula
Sodium chloride	NaCl	> 99.5	58.44
Magnesium chloride, hexahydrate	MgCl_2_·6H_2_O	> 99	203.30
Calcium chloride	CaCl_2_	> 99	110.98
Sodium sulfate	Na_2_SO_4_	> 99	142.04
Magnesium sulfate, heptahydrate	MgSO_4_·7H_2_O	> 99	246.48

**Figure 4:**
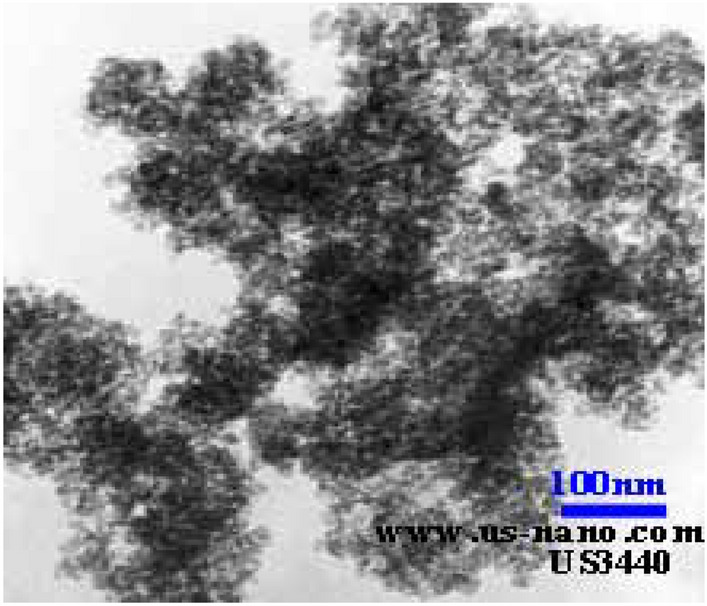
SEM analysis of nanoparticles used in experiments.

Silicon oxide nanoparticles with dimensions of 15–20 nm and 99.95% purity were purchased commercially (Fig. 4) (the reason for using silica nanoparticles for emulsion stability, in addition to the cases mentioned earlier, is because these nanoparticles are present in sand and clay formations that are not well packed and spontaneously in the production fluid). Span 80 surfactant is a non-ionic and lipophilic surfactant purchased from Sigma-Aldrich.

### Method of work

#### Making an aqueous solution

Initially, brine solutions were made using different salts (NaCl, Na_2_SO_4_, CaCl_2_, MgCl_2_, MgSO_4_) at concentrations (1000, 5000, 10,000, and 50,000 ppm) on a stirrer at 500 rpm. Then silica nanoparticle powder with different concentrations was added to each sample. The solution was then subjected to ultrasonic waves for 5 min to disperse the nanoparticles. Then, the surfactant was added in a certain amount, and the solution was homogenized for nine minutes at 9000 rpm with a homogenizer.

#### Emulsion preparation

A homogenizer was used to homogenize the crude oil and aqueous solution at 7000 rpm for eight minutes during emulsion preparation. In addition, hand-shaking was used to evaluate the ease of emulsification.

#### Viscosity measurement

In this study, two viscometers were used for viscosity measurement. (1) a rolling ball viscometer made by the same research team and (2) Anton Paar rheometer model MCR-302 were used to validate the viscosity model of the rolling ball viscometer. The rolling ball viscometer consists of a cylindrical tube filled with a sample fluid and can be measured by applying the desired pressure and temperature to the system. When the cylinder is positioned at the desired angle, the ball begins to accelerate, and after a while, the ball reaches a constant speed, which can use to calculate viscosity. If the flow around the ball is considered laminar, the viscosity can be obtained using the following equation:

1 $$\mu =K t \left({\rho }_{b}-{\rho }_{f}\right) sin\theta $$

ρ_b_: density of ball gr/cc, ρ_f_: density of fluid gr/cc, θ: the angle of inclination of the cylinder from the horizontal position (75°), t: Duration of the ball movement, second, $\upmu$: viscosity, K: constant of rolling ball viscometer.

#### Emulsion stability

In this paper, the emulsion stability has been investigated by using the bottle test analysis method. In this method, the stability of the emulsion is investigated by observing the volume of the separated phase. Pour 10 ml of each sample into a falcon and seal it with Teflon tape and keep it at room temperature for a month. During this period, the samples are imaged, and the phase separation is examined as a function of stability. In the optimal state, phase separation was not observed after 30 days.

## Results and discussion

There are several factors that affect the effective viscosity of water in oil emulsions, including the volume fraction of water, temperature, pressure, shear rate, mean droplet size, droplet size distribution, oil viscosity and density, interfacial tension between two phases, and emulsion stability.

### The impact of water content

**Figure 5:**
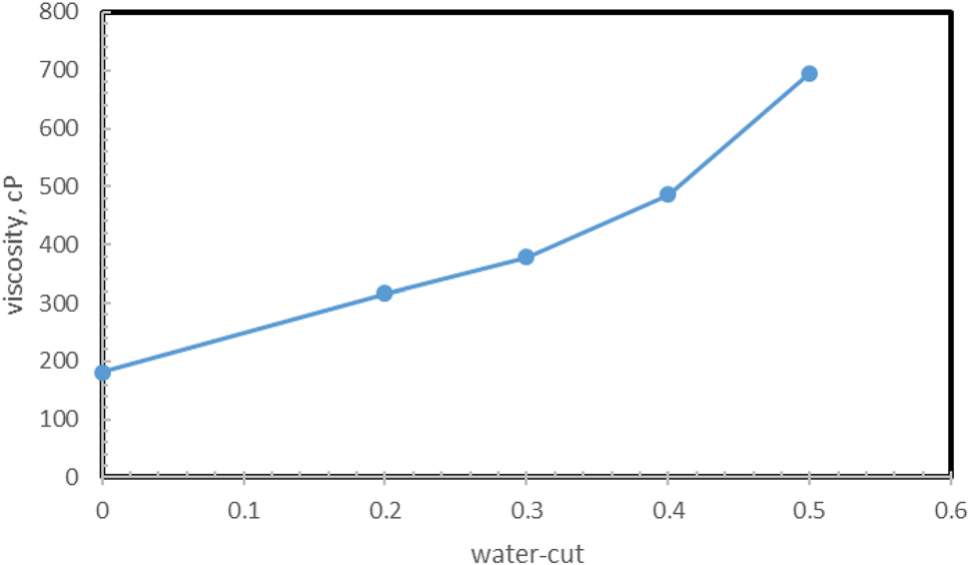
Effect of water-cut on the viscosity of the emulsion.

The effect of the water cut was examined at five levels, which include 0%, 20%, 30%, 40%, and 50%. Figure 5 illustrates the effect of water cut on the viscosity of water in crude oil emulsions.

As shown in Fig. 5, the apparent viscosity and relative viscosity of the emulsions increase as the water content increases. As the water cut increases, the number of water droplets in a certain volume increases, and the interaction of these droplets cause to change in the viscosity (Table 4). Asphaltene molecules interact with non-polar or hydrophobic groups to produce steric repulsion. Asphaltene molecules can extend their side chains significantly to the oil phase, and their steric repulsion can prevent aggregation of the bonds by maintaining a sufficient distance between them. Molecules on the surface of oil and water increase the surface viscosity and apparent viscosity of the oil in the film between the droplets. Both of these effects increase viscosity and prevent aggregation. In 2020, Du et al. conducted an experimental study to determine the effects of water in oil emulsions using nitrogen and carbon dioxide in the EOR process, which with increasing water cut, viscosity increases significantly. The viscosity increased up to 70% water cut, but then decreased due to the change of the emulsion type from a single W/O emulsion to an O/W/O composite emulsion. They explained that as the water cut increases, the number of scattered water droplets increases, and the distance between them decreases. The results showed that the stability and viscosity of emulsion increased. Increasing the amount of water to 70% reduces the number of droplets, but increases droplet size. As a result, the emulsion becomes more unstable, and the viscosity decreases^65^.

**Table 4:** Effect of water content on emulsion properties.

Variable parameters	Target parameters	effect	References
Water cut, temperature, and shear stress	Viscosity	Viscosity increases with increasing water-cut and decreases with increasing temperature and shear stress	^66^
Water cut, chitosan polysaccharide, and surfactant span 80	Average droplet size and emulsion viscosity	They showed that increasing the viscosity of the emulsion when increasing the amount of water from 5 to 35% results in finer droplets and a more uniform droplet distribution of emulsions	^67^
The water cut and amount of pectin	Emulsion viscosity	By increasing the amount of water in the range of 20–50%, the viscosity of the emulsion increases	^68^
Water cut	Emulsion viscosity	Emulsion viscosity increases significantly as the amount of water increases, but this increase was observed up to 70% of the water cut, but then As the type of emulsion changed from single W/O to double W/O, the viscosity decreased composite emulsion O/W/O	^69^
Water cut	Emulsion viscosity	As the water cut increases, the viscosity of the emulsion also increases	^70^
Water cut	Emulsion viscosity	Viscosity increases as dispersion volume increases. This is because, in the high water cut, there is a more considerable amount of water and a more significant number of water droplets per unit volume of the emulsion	^71^
Water cut	Emulsion viscosity	As the amount of water increases in the early times, the viscosity increases	^72^

### Effect of salt type and concentration

The density difference between the two phases based on the Gibbs adsorption isotherm directly affects the interfacial tension so that increasing the density difference increases the interfacial tension. Many papers have dealt with the effect of different cations on the stability and droplet size of the emulsion. In this article, five types of salts have been studied for emulsion prepration at 50% water cut (NaCl, MgCl_2_, CaCl_2_, Na_2_SO_4_, MgSO_4_).

**Figure 6:**
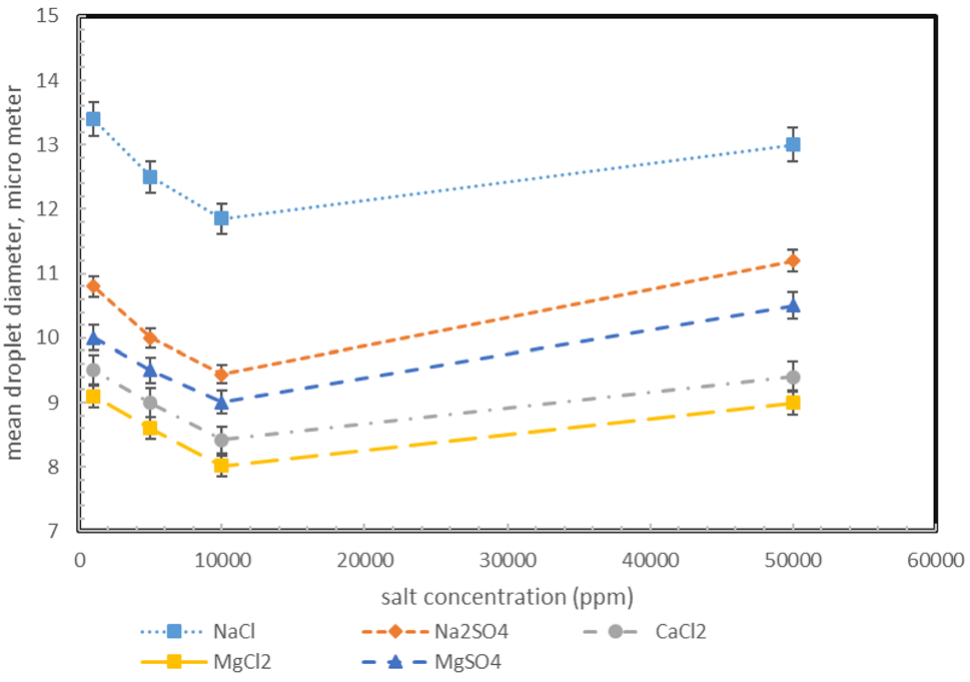
Effect of salt type on mean droplet size at 50% water content.

**Figure 7:**
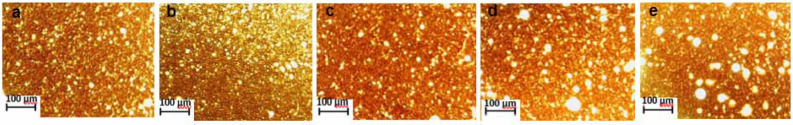
Effect of salt type on mean droplet size (**a**) MgCl_2_, (**b**) CaCl_2_, (**c**) MgSO_2_, (**d**) Na_2_SO_4_, (**e**) NaCl at 10,000 ppm.

As shown in Figs. 6 and 7, the size of the droplets at first decreases and then increases, an inverse relationship with the stability trend. However, viscosity shows a similar trend to stability^73–75^.

In the interaction of particles, there are two types of intermolecular forces: gravitational forces (Van der Waals), as well as repulsive (electric) forces. The presence of free cations and anions at the interface of water and oil causes an electric layer to form at the interface as an emulsion forms.As the salt crystal dissolves in water, it produces an electric charge, which increases the ionic strength. When the water in oil emulsion is formed, these ions are absorbed by the emulsion droplets and generate an electric charge, creating an electrical double layer that has an electrostatic repulsion. Two factors determine the tension of the second layer: the concentration of electrolyte and the capacity of the electrolyte. Having a low concentration and capacity makes the second layer longer. In other words, as the electrolyte concentration increases, the thickness of the layer decreases. Accordingly, repulsion forces decrease with increasing electrolyte concentrations. Emulsion stability depends on the force of attraction between the droplets. Higher ion charge density leads to shorter electrical layers. By comparing monovalent and divalent salts, cations with divalent charge density are observed to have a higher density than cations with monovalent charge. Consequently, a film is formed around the water droplets containing asphaltene particles, and the asphaltene is attracted to the water–oil interface through ions. As the asphaltene moves to the interface between the two fluids, the interfacial tension decreases, and the emulsion stability will increase. MgCl_2_ and MgSO_4_ salts were selected to investigate the effect of monovalent and divalent anions on emulsion formation and stability. Divalent sulfate anions are larger than chloride anions; it causes less asphaltene to be transferred to the interface of the aqueous phase. However, heteroatoms such as O^2-^, S^2-^, and N^2-^ are also present in asphaltene molecules, which has a charge equal to sulfate, makes the presence of asphaltene in the aqueous phase more limited. The lower presence of asphaltene in the aqueous phase interface causes a reduction in the interaction of cations with asphaltene molecules. Therefore, with the reduction of asphaltene in the interface between the aqueous and oil phases, the stability of the emulsion decreases. Therefore, as the anion charge increases, the stability of the water/oil emulsion decreases due to the reduction of the stabilizing agent (asphaltene) at the interface of the two phases. The results obtained in this study are consistent with those obtained in previous publications, and the effect of ions on the stability of emulsions can be classified as their effect on stability in the following manner: Mg^2+^ > Ca^2+^ > Na^+^, which is quite valid according to the results in the other articles Table 5. According to the cases that have been studied in the past, the order of efficiency of cations in emulsion stability and reduction of interfacial tension has been as follows, which confirms our results: Na^+^  < Li^+^  < Ca^2+^  < Mg^2+^^76,77^.

**Table 5:** Effect of salt on emulsion properties.

Salt type	Target parameters	Effect	References
NaCl	Emulsion stability	As the salinity increases, the IFT increases, and as a result, the larger droplets decrease the emulsion stability	^78^
NaCl	Effect of salinity on surface properties of emulsion	By increasing NaCl concentration, the viscosity and elastic modulus increase, the surface activity of surfactants increases, and the stability of emulsions increases	^79^
NaCl	Emulsion stability	Increasing the concentration of NaCl increases the activity of surfactant molecules at the oil–water interface, and this phenomenon reduces the droplet size of the emulsion and increases its stability by reducing the IFT	^80^
NaCl	Viscosity and stability of the emulsion	As the salt concentration increases, the average droplet size decreases, and the emulsion stability increases. Since there is a direct relationship between stability and viscosity, the viscosity will also increase	^81^
CaCl_2_	Emulsion stability	The average droplet size decreases as calcium chloride concentration increases and emulsion stability increases	^82^
CaCl_2_	Emulsion stability	Calcium ions increase the gravitational force between water droplets as their concentration increases, and the amount of surfactant adsorption on the surface increases, As a result, the droplet size decreases and the emulsion stability increases	^83^
CaCl_2_	Emulsion viscosity	The emulsion's viscosity increases with an increase in salt concentration. According to the results, these changes in low water content are not very significant, but with increasing water content, changes in emulsion viscosity increase significantly with increasing salt concentration	^84^
Na_2_SO_4_	Emulsion stability	Reducing the interfacial tension between the oil and brine provides the conditions for stable emulsions, stating that increasing the salt concentration will reduce the IFT more	^84^
NaCl, Na_2_SO_4_	Phase inversion	The effect of Na_2_SO_4_ salt is more than NaCl, and with increasing salt concentration, the phase inversion temperature decreases	^85^
Na_2_SO_4_	Emulsion stability	IFT is significantly reduced by adding sulfate ions to brine, and the emulsion stability and droplets size increase and decrease, respectively	^86^
Na_2_SO_4_, NaCl	Emulsion stability	Adding Na_2_SO_4_ salt to the dispersed phase will reduce the droplet size, and the emulsion stability will increase. They also stated that the effect of Na_2_SO_4_ and NaCl salts would be more than NaCl	^78^
MgCl_2_	Emulsion stability	Upon reaching a threshold concentration of ions, the stability of the emulsion increases and then decreases	^87^
MgCl_2_	Emulsion stability	By adding MgCl_2_ salt, the emulsion stability was higher than the initial state	^78^
MgSO_4_, Na_2_CO_3_	Emulsion stability	An increase in salt concentration results in more ion interactions with asphaltene, and as the droplets decrease, the stability increases. It was also noted that each of these salts has an optimal concentration to increase stability	^88^

**Figure 8:**
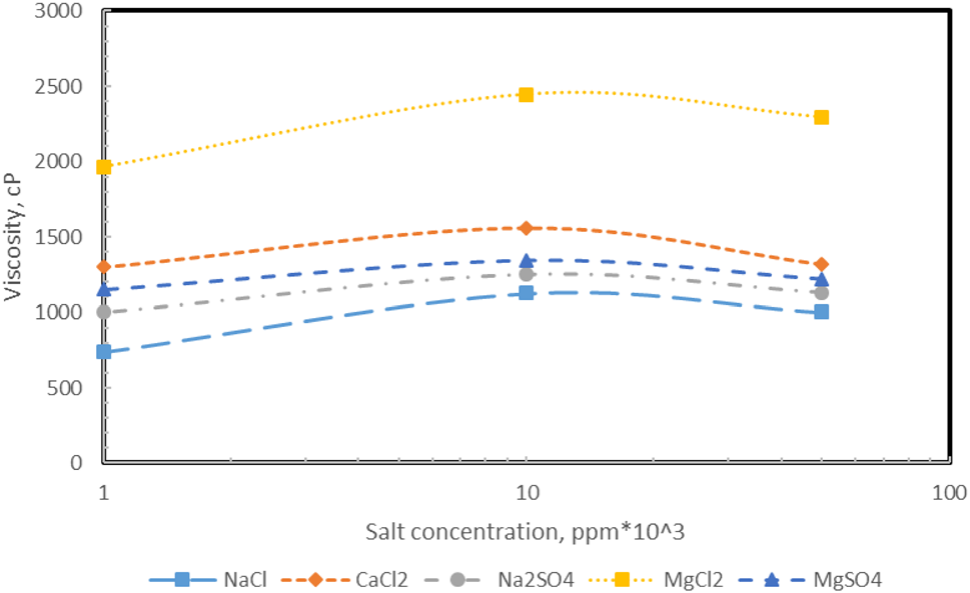
Effect of salt type and concentration on the viscosity of emulsions.

According to the results obtained, as shown in Fig. 8, the viscosity of the emulsion increases with increasing salt concentration to 10,000 ppm and then decreases. Based on the microscopic images, salt concentrations up to 10,000 ppm decrease droplet size and increase viscosity. However, larger droplets form after that concentration due to Ostwald ripening, and viscosity decreases due to the coating over the interface of emulsion droplets.

Ghannam studied the effect of changes in NaCl concentration on the properties of W/O emulsions. The results of the study indicated with increasing salt concentration, the average droplet size becomes smaller, and the stability of emulsions increases, and since there is a direct relationship between stability and viscosity, the viscosity will also increase^81^. Marquez et al. Investigated changes in emulsion stability in the presence of different concentrations of calcium salts. They stated that as the concentration of calcium ions increases, the gravitational force between water droplets should decrease, and the amount of surfactant adsorption on the surface increases. As a result, droplet size is reduced, and emulsion stability increases. This reduction in size increases the viscosity of the emulsion, which may reduce the accumulation rate of water droplets^83^. Gomez examined the effect of CaCl_2_ salt concentration and oil type on emulsion viscosity and showed that with increasing salt concentration, the emulsion viscosity increases. According to the results, these changes in low water content are not very significant, but the difference in viscosity with salt concentration increases significantly with increasing water content. The presence of higher ionic strength will cause the polar molecules in the oil to move towards the two-phase interface and form smaller droplets. With smaller droplets, the surface area of the droplets increases, and the viscosity of the fluid increases^84^. According to the results, droplet size reduction is directly correlated with the viscosity of the emulsion. As a result, natural surfactants move more toward the interface of the two phases as salt concentration in the aqueous phase increases due to the number of ions. This phenomenon causes the droplet size to become smaller to a threshold concentration, but after that, the droplets coalesce due to the Ostwald ripening phenomenon, and their average size increases. As the droplet size decreases and then increases, the total viscosity increases and decreases, respectively^89^.

### Effect of nanoparticles and surfactants

**Figure 9:**
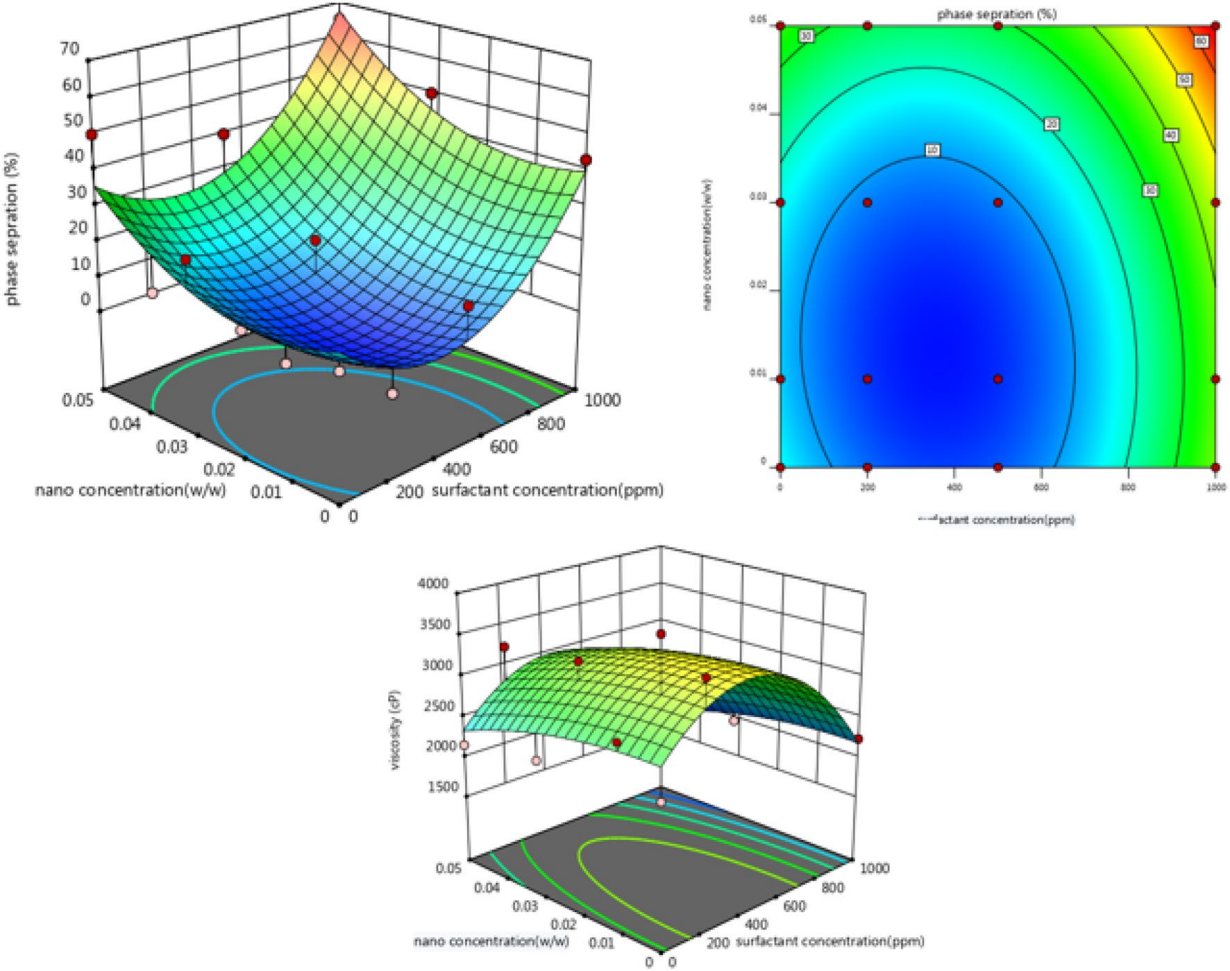
Effect of nanoparticle and surfactant concentration on viscosity and phase separation after 30 days (3D and contour plots).

Emulsifiers often stabilize emulsions used in the EOR process. Emulsifiers reduce interfacial tension and thus increase the emulsion stability by transferring toward the oil and water interface^10^. Surfactants molecules can be thickening the oil phase by small emulsions flocculation. This phenomenon increases the rigidity of the surface of the emulsion droplets and increases the stability of the emulsion. In addition, Silicon oxide nanoparticles facilitate the adsorption of surfactant molecules by creating a mechanical barrier effect. In general, in the injection of surfactant alone in the EOR processes, the amount of surfactant in the water phase decreases because of the surfactant's high adsorption on the rock surface, and the interfacial tension between oil and water increases^19–21^. Nanoparticles increase emulsion stability through adsorption on the surface of water and oil. As a result, coagulation, flocculation, and droplet coalescence are minimized by this process^30^. However, the use of nanoparticles alone has limitations for the injection process due to their easy and rapid aggregation. Therefore, using each of these factors alone has limitations during the EOR processes. Nevertheless, the synergistic effect of combining nanoparticles with surfactants can cause more potent properties than using these two materials alone. Simultaneous use of these two with MgCl_2_ salt can increase the performance efficiency of nanoparticles and surfactants for the EOR processes^18^. With the presence of nanoparticles in the aqueous phase, the rate of surfactant loss due to surface coverage by nanoparticles in whole or in part is reduced, and also surfactants can bring the nanoparticles to the interface of oil and water through Brownian motion and cause further reduction in interfacial tension^22^. However, the absence of a surfactant also causes the nanoparticles to coalesce rapidly due to the high energy level (due to the high surface-to-volume ratio), and due to this rapid accumulation, stability is lost^23,24,28^. Surfactants can induce the same charge by repulsing the nanoparticles and causing them to disperse more^90^. The concentration of nanoparticles and surfactants has an optimal concentration. Increasing the concentration of nanoparticles and surfactants increases the stability and viscosity of the emulsion, and decreasing concentrations causes stability and viscosity to decrease. This phenomenon reduces the interface between the two phases, the viscosity, and the emulsion stability (Fig. 9).

**Figure 10:**
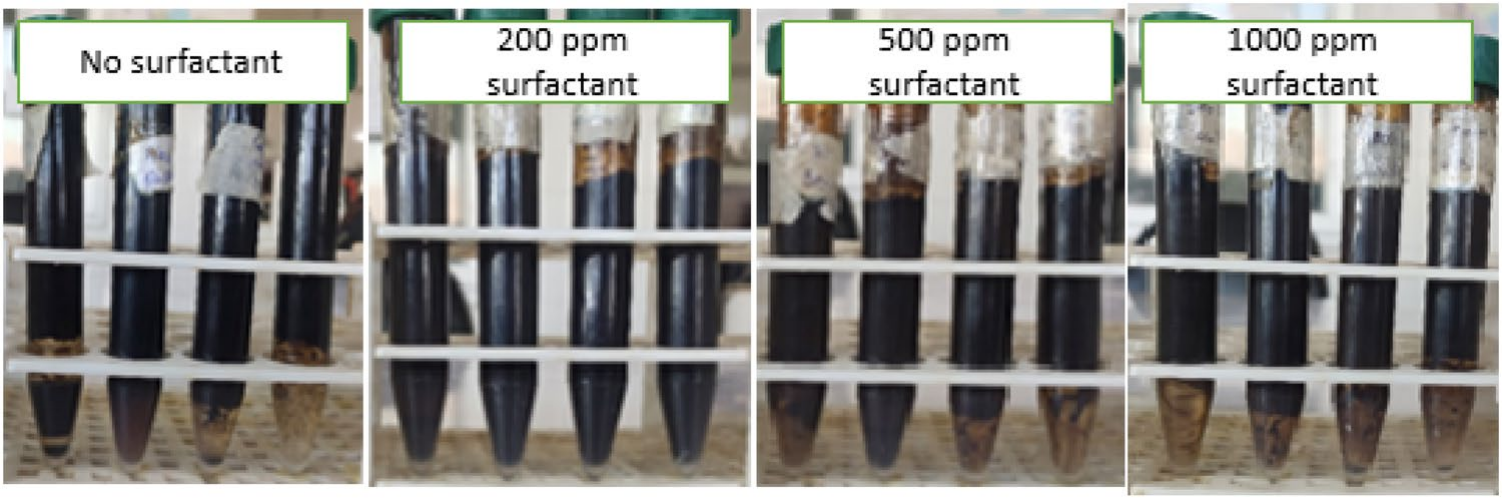
Phase separation after 30 days (0%, 0.1%, 0.3%, 0.5% (w/w) concentration of nanoparticle and 10,000 ppm concentration of MgCl_2_ from left to right in each case).

According to the results obtained from the bottle test (Fig. 10), the optimal concentration of nanoparticles in all samples was 0.1% (w/w), and the optimal concentration of surfactant was 200 ppm. The combination and synergy of nanoparticles with surfactant and available salts causes more stability due to enhancing the mechanical barrier and reducing the rate of aggregation of droplets in emulsions containing nanoparticles, surfactant, and salt against conventional emulsions or emulsions with surfactant. Therefore, the stability of the emulsions produced in this study is increased by the ionic strength of the salt, Reduction of IFT and rigid film formation by small emulsions due to the presence of surfactant, and by the formation of a strong mechanical barrier with silicon oxide nanoparticles. The results obtained in this study are similar to Nesterenko's observations^91^.

**Figure 11:**
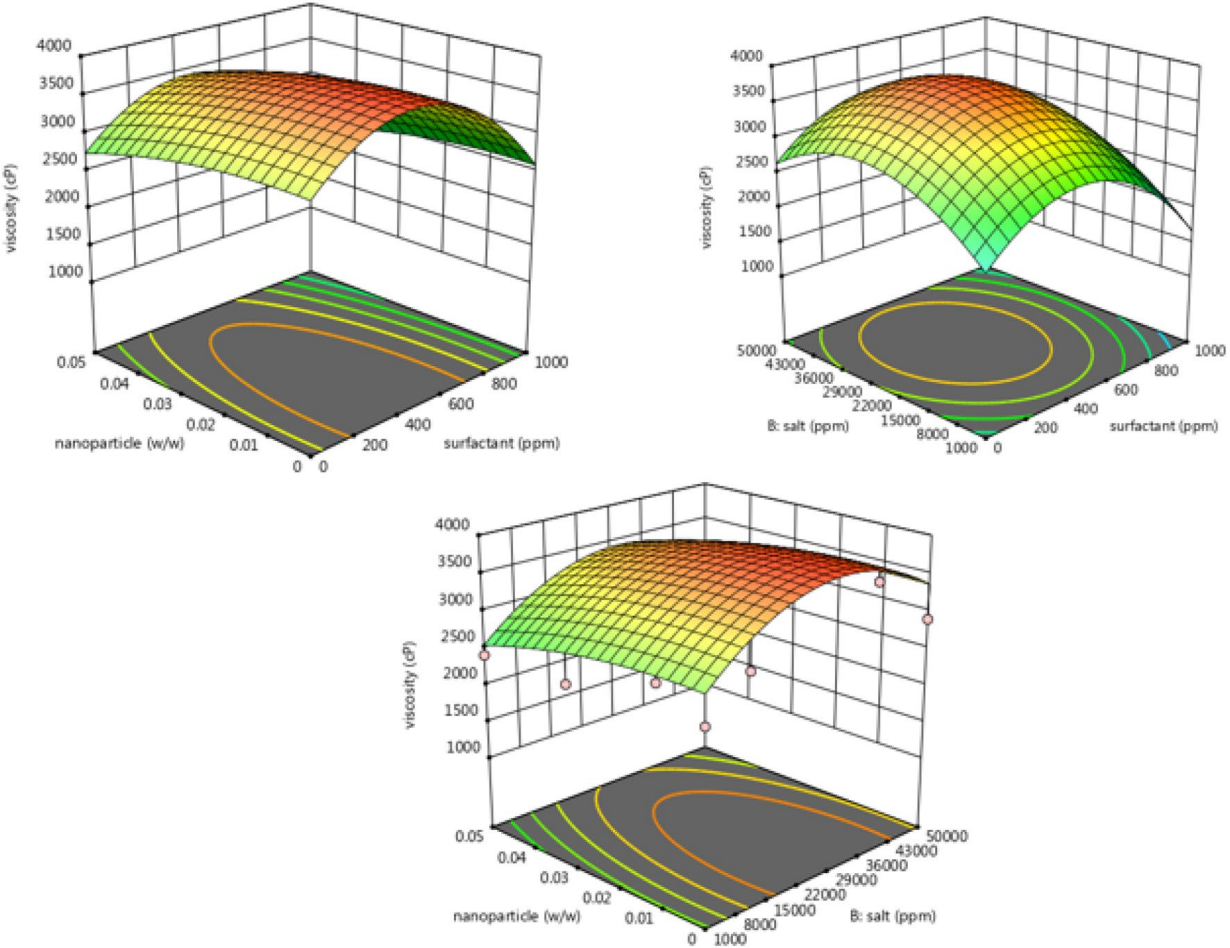
Effect of surfactant and nanoparticle concentrations in the presence of various salt concentrations on viscosity.

Changes in the rheological properties of emulsions can change the number of emulsion droplets colliding and affect stability. As can be seen in the Fig. 11, in addition to enhancing emulsion stability, the rheological behavior of the emulsion must also be improved, indicating a direct relationship between viscosity and stability^92^.

One of the parameters that greatly affect viscosity is droplet size. The surface area of the droplets determines the friction between them. In other words, when the ratio of area to volume of droplets increases, viscosity increases. Therefore, an emulsion with a smaller average droplet size has greater apparent viscosity. When the emulsion has a high viscosity and emulsification takes place through mixing, the use of shear force is more effective and results in much smaller droplets. It should be noted that little is known about how the size of droplets influences emulsion viscosity in the literature^70,93–95^.

### Effect of nanoparticles and surfactants on emulsification

**Figure 12:**
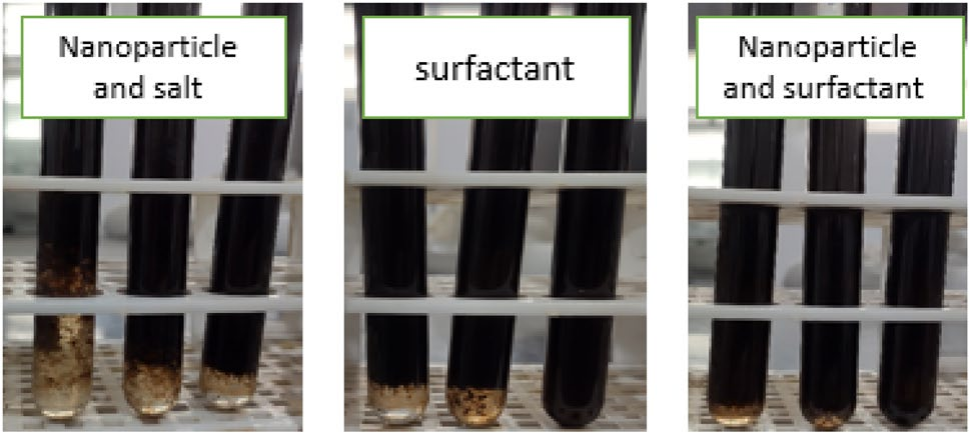
Emulsification after 10, 20, and 25 times shaking from left to right.

This paper uses the handshake method to evaluate the emulsification ability. The results show that the emulsification in the sample containing the surfactant is easier than in the sample without the surfactant (Fig. 12). In this study, samples with surfactants and samples with both surfactants and nanoparticles were emulsified easier than samples that did not have surfactants. In the case of samples where the surfactants were used alone or in combination with the nanoparticle, the emulsion formed after 25 times diverting if the samples without the surfactant did not show this behavior.

According to Gibb’s free energy, as systems naturally tend to reduce energy, emulsions that have more free energy are more difficult to form. When the free energy is low, the ability of emulsification will be greater, and the emulsion formation will be easier. According to the definition of interfacial tension (energy per unit of length), the concept of free energy is closely related to this parameter. If the interfacial tension in an emulsion system decreases, the required energy for emulsification will decrease.

Zhao et al. In 2020 also showed that emulsification is easier due to the presence of surfactants in the system. They observed this phenomenon using the number of shaking tims required for emulsification. They found that the number of shaking times for emulsification in systems with the surfactant was much lower than in systems without surfactant^96–99^.

## Conclusion

This study aimed to investigate the viscosity behavior and emulsion stability in the presence of NaCl, CaCl_2_, MgCl_2_ and Na_2_SO_4_ salts, different salt concentrations, different water cut, span80 surfactant, and silicon oxide nanoparticles. According to the experiments performed on the emulsions, the following results were obtained:

1. With an increase in water content, droplets become smaller, resulting in a larger interface and the accumulation of intrinsic surfactant molecules on both water and oil surfaces, and the electrostatic repulsion force of the electric double layer increases. Consequently, the droplet volume decreases and the emulsion viscosity increases.
2. Regarding the use of different salts, as observed, MgCl_2_ > CaCl_2_ > Na_2_SO_4_ > NaCl demonstrated the best performance in terms of reducing droplet size, respectively. As a result, MgCl_2_ and CaCl_2_ showed the best performance among the salts.
3. Viscosity showed a similar trend with droplet size data. As the average droplets size decreased, the viscosity of the emulsion increased, which was consistent with the literature results.
4. Because divalent sulfate anions are larger than chloride anions, it reduces the amount of asphaltene that is transferred to the aqueous phase. Alternatively, because asphaltene has heteroatoms, such as O^2−^, S^2−^, and N^2−^, in its structure, which has a charge equal to sulfate, limits its presence in aqueous solution. Therefore, W/O emulsions become less stable as anion charge increases due to the reduction of the stabilizing agent (asphaltene) at the interface of the two fluids.
5. Monovalent cations have a lower charge density, as a result of which a thin film forms around charged asphaltene particles in water droplets, and asphaltene tends to be adsorbed by the existing ions between water and oil. The asphaltene is moved to the interface of the fluids, thus decreasing the interfacial tension and increasing the emulsion stability.
6. With the use of nanoparticles and surfactants for greater stability, it should be noted that surfactants have significantly reduced the energy required for emulsification. These results were obtained from the handshake method, which formed an emulsion after 25 times shaking the samples containing surfactant.
7. The stability of emulsions has increased during the simultaneous use of nanoparticles and surfactants, and this is due to the synergistic effect of using these two materials by creating a mechanical barrier with nanoparticles and electrostatic repulsion of existing surfactants and cations.
8. According to the results obtained from stability and viscosity tests, it was shown that the best water cut is 50% (due to higher viscosity and stability). MgCl_2_ showed the best performance in increasing viscosity and stability, and its optimal concentration was 10,000 ppm. Finally, by using the bottle test and evaluating the emulsion viscosity, the maximum stability and viscosity are related to the concentration of nanoparticles of 0.1% and surfactant at 200 ppm.

## Data Availability

All data generated or analysed during this study are included in this published article.
